# Fussy eating in toddlers: A content analysis of parents' online support seeking

**DOI:** 10.1111/mcn.13171

**Published:** 2021-03-19

**Authors:** Kylie Fraser, Brittany Reese Markides, Norma Barrett, Rachel Laws

**Affiliations:** ^1^ School of Health and Social Development Deakin University Victoria Australia; ^2^ Institute for Physical Activity and Nutrition (IPAN), School of Exercise and Nutrition Sciences Deakin University Victoria Australia

**Keywords:** diet, food refusal, fussy eating, online forums, parental feeding practices, picky eating, toddlers

## Abstract

The development of healthy eating habits in childhood is essential to reducing later risk of obesity. However, many parents manage fussy eating in toddlerhood with ineffective feeding practices that limit children's dietary variety and reinforce obesogenic eating behaviours. Understanding parents' feeding concerns and support needs may assist in the development of feeding interventions designed to support parents' uptake of responsive feeding practices. A total of 130 original posts by parents of toddlers (12–36 months) were extracted from the online website Reddit's ‘r/Toddlers’ community discussion forum over a 12‐month period. Qualitative content analysis was used to categorise the fussy eating topics that parents were most concerned about and the types of support they were seeking from online peers. The most frequently raised fussy eating concerns were refusal to eat foods offered, inadequate intake (quantity and quality), problematic mealtime behaviours and changes in eating patterns. Parents were primarily seeking practical support (69.2%) to manage emergent fussy eating behaviours. This consisted of requests for practical feeding advice and strategies or meal ideas. Nearly half of parents sought emotional support (47.7%) to normalise their child's eating behaviour and seek reassurance from people with lived experience. Informational support about feeding was sought to a lesser extent (16.2%). Fussy eating poses a barrier to children's dietary variety and establishing healthy eating habits. These results suggest parents require greater knowledge and skills on ‘how to feed’ children and support to manage feeding expectations. Health professionals and child feeding interventions should focus on providing parents with practical feeding strategies to manage fussy eating. Supporting parents to adopt and maintain responsive feeding practices is vital to developing healthy eating habits during toddlerhood that will continue throughout adulthood.

**Key messages**

- Parental concerns about fussy eating in toddlerhood are common, and many parents manage fussy eating with counterproductive feeding practices for developing healthy eating habits in early childhood.
- Parents are seeking practical and realistic advice in online forums for managing the emergence of fussy eating behaviours.
- Nutrition training for health professionals, including maternal and child health nurses and doctors, should have greater emphasis on ‘how to feed’ using responsive feeding approaches that support the development of healthy eating habits.

## 1 INTRODUCTION

Early childhood is a crucial period for the development of healthy eating behaviours (Adamo & Brett, 2014). Parents play an important role in the development of children's early food preference and acceptance patterns (Birch & Fisher, 1998). Children's exposure to a variety of nutritious food, particularly fruit and vegetables, can promote healthy dietary habits and reduce the risk of obesity (Birch & Anzman‐Frasca, 2011). However, many children in high income countries consume insufficient amounts of fruits and vegetables. Only 7% of children aged 2–18 years in the United States (US) consume the recommended daily intakes of vegetables (Nekitsing et al., 2018), while less than 13% of Australian children aged 2–4 years and 18% of 5–18 year‐olds in the United Kingdom (UK) consume adequate portions of fruits and vegetables (Australian Bureau of Statistics, 2018; Nekitsing et al., 2018). In addition, global obesity rates have increased rapidly in children under the age of 5 years (WHO, 2020), with childhood obesity predictive of obesity in adulthood (Singh et al., 2008). Therefore, parental support to develop healthy eating behaviours from an early age is paramount to shaping life‐long eating habits.

The emergence of fussy eating during toddlerhood can contribute to problematic parent–child feeding interactions (Walton et al., 2017). ‘Fussy’ or ‘picky’ are used to describe a spectrum of food refusal behaviours including the refusal of both familiar and unfamiliar foods (neophobia), limited dietary variety and inadequate intake (Brown et al., 2016). Although a common and transient phase of child development, fussy eating is reported by up to 59% of parents as a source of concern (Brown et al., 2016). A recent Australian survey of parents (*n* = 330) of young children (2–5 years) identified fussy eating as the main feeding concern for over half (53.9%) the respondents (Boswell et al., 2019). Furthermore, problematic feeding behaviours are frequently associated with high levels of maternal distress and mealtime conflict (de Barse et al., 2016; Harris et al., 2018; Jarman et al., 2015; Trofholz et al., 2017).

The relationship between child–parent feeding interactions is complex and widely recognised as bi‐directional in nature (Jansen et al., 2017; Mallan et al., 2018). Many parents misinterpret developmentally normal fussy eating behaviours as problematic and respond with non‐responsive feeding practices such as pressure, catering to preferences and using food as a reward. These practices have been shown to promote higher food fussiness (Mallan et al., 2018) and promote obesogenic eating behaviours (Rodgers et al., 2013). Conversely, responsive feeding practices such as repeated exposure, modelling and structured mealtimes have been associated with more desirable eating behaviours and self‐regulation of energy intake (Daniels, 2019; Finnane et al., 2017; Magarey et al., 2016).

Tailoring interventions specifically to address common feeding concerns encountered during toddlerhood is important for engaging parents (Boswell et al., 2019). Yet there is a dearth of research investigating the specific fussy eating behaviours parents perceive to be problematic and their support seeking behaviour. Quantitative studies have relied on pre‐defined lists of typical fussy eating behaviours which prevents the open exploration of parents' experiences and unanticipated responses (Boswell et al., 2019; Taylor et al., 2015). Additionally, existing research has been primarily conducted with focus groups or surveys which are subject to social desirability bias and rely on retrospective reports or have used small samples of parents seeking professional support for extreme fussy eating concerns (Harris et al., 2018).

Increasingly, parents, particularly mothers, are utilising online mediums for parenting information, advice and social support (Moon et al., 2019). A recent study which recruited mothers through a parenting Facebook group identified that online forums were consulted for fussy eating information and support almost as often as health professionals (Curney & Wilkinson, 2016). An exploration of the fussy eating concerns parents' report online may capture broader perspectives of common feeding concerns while minimising social desirability bias. Additionally, an exploration of the types of support parents want/need to manage these concerns has potential to inform the development of interventions to maximise uptake and effectiveness (Peters et al., 2013).

The present study seeks to gain a deeper understanding of parents' first‐hand experiences of fussy eating in toddlers by analysing posts on a popular online discussion forum. Specifically, the study aims to (i) identify the fussy eating concerns of parents with toddlers and (ii) classify the types of support parents are seeking in response to their feeding concerns. This knowledge is important for informing family health services and designing interventions which support parents to develop their child's healthy eating behaviours using responsive feeding practices during normal and transient phases of child development.

## 2 METHODS

### 2.1 Study design

The research project was situated within the constructivist paradigm (Mackenzie & Knipe, 2006) and adopted a qualitative descriptive approach as a methodological framework (Sandelowski, 2000). To understand parents' concerns and support seeking in relation to fussy eating, this study purposely identified an online social networking forum dedicated to parents of toddlers (12–36 months). The research process was underpinned by the constructivist positioning of subjectivity, which acknowledges the experiences of both the researcher and participants (Bradshaw et al., 2017). As a mother of two young children, the first author (KF) approached the data analysis phase from a position of ‘shared experience’ (Berger, 2015). As an ‘insider’, the researcher benefited from understanding the nuances of participants' experiences with fussy eating (Berger, 2015). Researcher reflexivity was central to recognising the first author's active role in the interpretation of participant's views. A journal was maintained by KF throughout the analysis phase to record and reflect on any preconceived ideas or thoughts to establish reflexivity and improve rigour (Mills & Birks, 2014). COREQ guidelines (Tong et al., 2007) have been adhered to in the reporting of this study.

### 2.2 Data source

Reddit is an online social media platform that allows users to upload news, media content, information and anecdotes into subject specific forums or communities called subreddits (‘r/subjects’) (Chew et al., 2019). Within subreddits, participants can post questions or join conversations (‘threads’) on a shared topic of interest by responding to an original post. Reddit is the 18th most visited website worldwide, hosting over 430 million monthly users globally (Alexa, 2020). The primary users of Reddit reside in United States (49.76%), United Kingdom (8.1%), Canada (7.59%), Australia (3.74%) and Germany (3.06%; Statista, 2020). All content posted on Reddit is publicly available and provides anonymity through the use of usernames or pseudonyms (Reddit, 2020).

### 2.3 Data collection

**FIGURE 1:**
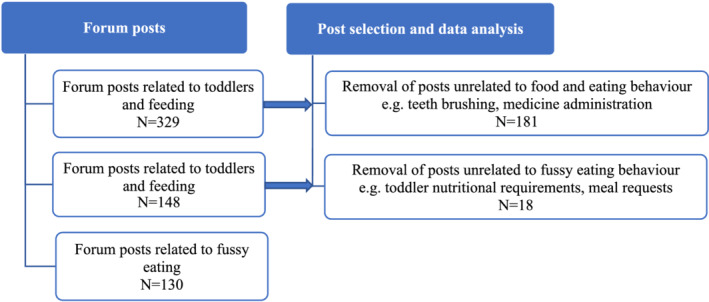
Selection of posts for analysis from Reddit r/Toddlers forum

The ‘r/Toddlers’ community was identified as the most relevant subreddit for analysing posts relevant to fussy eating in toddlers, as there are no specific subreddits on this topic. Over 105,000 parents subscribe to the r/Toddlers community and participate in 481 conversations (‘threads’). Data collection and preparation were performed by BRM using Python and Structured Query Language (SQL) programs created and executed in Jupyter Notebooks (Kluyver et al., 2016). First, all posts made on the r/Toddlers subreddit between August 2018 to September 2019 (3,069 posts) were extracted from the publicly available Pushshift Reddit dataset (Baumgartner et al., 2020). Then, to identify posts related to food and eating, topic modelling was performed using the Natural Language Took Kit (Bird et al., 2009) and Gensim Python libraries (Rehurek & Sojka, 2010). Original posts were retained for analysis as this study was focused on the support seeking practices of parents presenting with fussy eating concerns, yielding 329 posts, which were exported to Microsoft Excel for further refinement (Figure 1). Of these posts, 130 were related to fussy eating based on existing literature definitions of common food fussiness behaviours (e.g., food refusal and limited liked foods; Taylor et al., 2015; Wolstenholme et al., 2020). Data retrieved included post date and time, user pseudonym and the original post title and body.

The first author screened and refined all original posts (*n* = 329) for relevance to the research question. Co‐authors reviewed and verified posts excluded due to lack of relevance to eating behaviour and relatedness to fussy eating.

### 2.4 Data analysis

A qualitative content analysis using both inductive and deductive approaches (Graneheim et al., 2017) was utilised to identify (i) topics of concern and (ii) types of support sought by parents regarding fussy eating behaviours. After reading the data several times and becoming familiar with the content, data analysis was conducted by the first author in two separate phases as detailed below using NVivo 12 qualitative analytic software to assign and organise codes. After initial coding, the dataset was imported into Microsoft Excel to enable quantification of data via filtering functions.

Topics of concern parents raised within their online forum posts were categorised inductively using open coding. As each post was read, all topics of concern related to fussy eating behaviours were identified as meaning units and assigned an initial code. Some posts contained more than one topic of concern. As new codes were developed, they were applied to the entire dataset. These codes were then condensed into categories by grouping together similar topics related to the same concern (Erlingsson & Brysiewicz, 2017).

To categorise the types of support parents sought regarding fussy eating, a deductive approach to content analysis was utilised (Elo & Kyngäs, 2008). After a preliminary examination of all posts, a modified version of Sillence's (2013) typology of advice‐solicitation was used to code the kinds of questions asked. The typology consisted of four categories: request for advice, request for opinion or information, problem disclosure and ‘anyone in the same boat’. This typology was chosen for the simplicity of the advice‐solicitation categories in classifying online support seeking questions. The principles of inductive content analysis were then used to create further categories for questions that did not fit within the coding framework (e.g., explicit requests for meal ideas; Elo & Kyngäs, 2008). Further abstraction of the data was conducted by reviewing the dataset with these categories in mind to obtain a sense of ‘what's going on’ (Elo & Kyngäs, 2008).

To increase the trustworthiness of findings, regular debriefings were conducted with the research team to provide varying perspectives and points of view for interpreting the data (Erlingsson & Brysiewicz, 2017). Prolonged engagement with the data, cross‐checking of excluded posts and authenticating participants' experiences using direct quotations within the findings were additional strategies employed to ensure research credibility (Bradshaw et al., 2017).

### 2.5 Ethical considerations

This study was granted an Exemption from Ethics Review by the Deakin University Human Research Ethics Committee (reference 2020‐144) in line with National Statement on Ethical Conduct in Human Research (NHMRC, 2007).

## 3 RESULTS

A total of 130 original posts by 121 unique users were analysed, which averaged a median length of 128 words. Seven users submitted two posts and one user submitted three separate posts within the 12‐month sampling period. Most posts consisted of the disclosure of a problem or sharing of a specific experience followed by the request for information, advice or support. Parents often described their experience using anecdotes, and two thirds (66.2%) expressed high levels of emotion such as concern, stress or frustration. Expressions of emotions were explicitly stated or inferred (e.g., using multiple exclamation marks). Almost one third (29.2%) of parents explicitly referred to either their child or child's eating behaviour as picky/fussy. The remaining posts (70.8%) described eating behaviours indicative of common fussy eating characteristics (e.g., food refusal and limited intake).

### 3.1 Topics of concern

The fussy eating topics most frequently raised in the r/Toddlers forum were categorised by food refusal (*n* = 102 posts, 83.1%), inadequate intake (*n* = 96 posts, 73.9%), problematic mealtime behaviours (*n* = 62 posts, 47.7%) and change in eating patterns (*n* = 53 posts, 40.7%). Each post often contained more than one fussy eating concern (Table 1).

**TABLE 1:** Fussy eating topics of concern

Category	Sub‐category	N (%)	Representative post excerpts
Food refusal	Refusal to eat/drink	78 (60%)	‘I've spent too much money going to the doctor for them to tell me what I already know. She. Won't. Fucking. Eat. It.’ #22
	Refusal to eat certain food groups: vegetables, fruits or proteins (meat/eggs)	24 (18.5%)	‘My 16 month old all of sudden stopped eating meat or any protein I try and give him really’. #37 ‘But when she sees there's a vegetable on her plate she goes completely crazy and refuses to eat ANYTHING’. #39
	Refusal of new foods (neophobia)	6 (4.6%)	‘My problem is when I offer my son new food he just says no and pushes it away. He won't even try it’ #23
Inadequate intake	Inadequate dietary variety	41 (31.5%)	‘I'm worried that there's not enough variety in her diet’ #123 ‘My two year old won't eat anything except the ten or so “approved foods …”’ #78
	Inadequate food/drink (milk or water) consumed	34 (26.2%)	‘I've always been frustrated with her eating because I feel it's so inconsistent and I feel like she basically just survives on air most of the day. Very rarely will she eat full meals’ #31
	Concerns about child growth	21 (16.1%)	‘He is so thin and the doctors have said it the past he will eat when he is hungry basically don't worry about it. We have tried this and he is just getting weaker’ #91
Problematic mealtime behaviours	Difficult behaviours: yelling, crying, gaging, throwing food and spitting	37 (28.5%)	‘… he literally slaps the spoon out of our hands or throws it on the floor while dramatically turning his face away and yelling’. #112
	Oppositional mealtime behaviour (e.g., refusing to sit at the table)	15 (11.5%)	‘My toddler won't feed herself even though she can pick things up with her hands she refuses. If me or her dad didn't feed her she wouldn't eat’. #18
	Slow eating	6 (4.6%)	‘Anyone else have a slow eater? … My kid stalls over food and won't focus at mealtime. Sometimes takes an hour to finish!!!’ #43
	Storing food in cheek	4 (3.1%)	‘My three year old held a partially masticated bite of cereal in her mouth for over an hour. I kept thinking she would surely swallow it. She did not’. #130
Change in eating patterns	Sudden and unexpected fussiness	33 (25.4%)	‘We try new foods almost daily, but he refuses to even try them. He wasn't always like this. There was a time he was basically a vacuum cleaner and would eat anything we put in front of him’. #111
	Frequent changes in liked, favourite or accepted foods	9 (6.9%)	‘We offer a variety of food but he's refusing basically everything, and stuff he used to like he won't eat now’. #51
	Inconsistent daily eating patterns	6 (4.6%)	‘If I heat up 4 chicken nuggets, my two year old will eat all 4, plus a Cutie orange, and still be hungry. If I heat up 5 chicken nuggets, she will eat 2 and a half and then be done’. #62
	Food/drink accepted in different contexts (e.g., daycare)	5 (3.8%)	‘My toddling adventurer goes to daycare 2 days a week, and there he eats almost everything they serve …. At home, if and when I serve him the same food, he'll just go “no” and toddle away and play’. #1

*Note*: Each post may contain more than one type of concern.

The fussy eating concern most frequently raised by parents was children's refusal to eat or drink foods offered (60%). Aversions to specific food groups (18.5%), namely, protein (meat and/or eggs), fruit and vegetables, were discussed more often than refusal of new foods (4.6%). Refusal to eat was often associated with additional feeding concerns such as challenging mealtime behaviours or nutritional quality and quantity of children's food intake. Ongoing refusal was a great source of anxiety and concern for some parents, even when they demonstrated an awareness of food preference development, such as the importance of repeated exposure.

I always offer and don't push her, I never force feed her or anything, and I know some kids need a lot of exposure before they start eating new foods. But how long?? #15

Concern about the nutritional quality and quantity of a child's dietary intake was expressed in nearly three quarters (73.9%) of parents' fussy eating concerns. Concerns for limited dietary variety (31.5%) were mostly related to a lack of fruit and vegetable intake. Many parents perceived their child's food intake as inadequate (26.2%), reporting ‘they do not eat enough’. Some parents were sceptical of their child's ability to regulate their own hunger, often referring to their child being ‘obviously hungry’. Lack of weight gain due to inadequate intake caused considerable concern for 21 parents (16.1%) who feared their child would ‘starve’ if they did not eat something. Some parents expressed these concerns despite direct acknowledgement that the child's doctor did not share the same concerns.

Most parents appeared to be conscious of the importance of providing a varied and balanced diet with many reporting they offered ‘healthy foods’. However, concerns for the perceived growth or nutritional impacts of limited intake or variety resulted in many parents tolerating the consumption of less nutritious and preferred foods. This was despite some parents' acknowledgement of the potential undesirable impacts of catering to children's preferences.

Almost one third (28.5%) of parents described the emergence of problematic behaviours when offering their child non‐preferred foods. Parents experienced a range of behavioural manifestations including verbal (e.g., saying ‘No’ and gagging) and non‐verbal gestures (e.g., pushing plate away, spitting food out and throwing food on the floor), to ‘full blown’ tantrums and meltdowns (e.g., kicking, screaming and yelling). These behaviours contributed to negative mealtime climates and conflict which some parents conceptualised as ‘dinner time battles’. In some instances, parents described using non‐responsive feeding practices including bribery or distractions (e.g., T.V.) as an act of desperation to reduce mealtime stress. Others stopped offering non‐preferred foods (e.g., vegetables) or used indulgent feeding practices such as catering to preferences or offering rewards (e.g., dessert and T.V. time) to prevent challenging mealtime behaviours. Although catering to children's preferences was a frequently mentioned strategy, several parents revealed that cooking separate meals resulted in increased stress. Additionally, several parents acknowledged the ineffectiveness of coercive or indulgent feeding practices in influencing their child's fussy eating behaviours.

Other parents expressed their frustration at their child's oppositional mealtime behaviours (11.5%). In these posts, children asserted control over feeding situations by refusing to do things for themselves (e.g., independent feeding or holding a bottle), refusing to sit at the table or requesting specific foods then refusing to eat them. Some parents attributed their child's challenging fussy eating behaviours to personality traits, referring to their child as ‘stubborn’. Slow eating (4.6%) and unusual eating behaviour, such as storing food in cheek (3.1%), were additional mealtime behaviours that concerned a minority of parents who found them inconvenient or frustrating.

Changes in toddlers eating patterns was identified as a cause for concern in two fifths (40.7%) of all posts. Parents were primarily concerned and frustrated by sudden or unexpected fussy eating behaviours (25.4%). A small percentage of parents (3.8%) expressed their frustration at their child's willingness to eat/drink in different contexts, namely, daycare. Others were confused or frustrated by the irregularity of the amount (4.6%) or types of food (6.9%) their child consumed from day to day. In some instances, parents acknowledged that their child's sudden or frequent fussy eating behaviour was most likely due to a developmental phase; however, they were still seeking emotional support from parents with lived experience.

### 3.2 Types of support sought

Of the 130 posts, 89% (*n* = 116) sought some type of support for fussy eating concerns. The main types of support parents sought within the forum were identified as practical support (*n* = 90 posts, 69.2%), emotional support (*n* = 62 posts, 47.7%) and informational support (*n* = 21 posts, 16.2%; Table 2). Some posts contained more than one advice‐solicitation request, and each request was categorised under a type of support.

**TABLE 2:** Types of support sought for fussy eating concerns

Main types of support sought and subcategories	Description	Frequency n (%)	Representative post excerpts
Practical support		90 (69.2%)	
Practical advice, tips, and strategies	Asks how/what one “should” do, or how/what “other” people do in a given situation	61 (46.9%)	‘… what do I do when he doesn't eat and is hungry later? Do I give him his uneaten dinner? Do I make him a different snack?’ #17
Practical meal ideas	Request for easy, toddler approved recipes	29 (22.3%)	‘Fussy toddler please spam with recipes!’ #74
Emotional support		62 (47.7%)	
Normalisation	Asks if situation is normal or if others are experiencing the same thing	28 (21.5%)	‘Is this normal? Do I need to do something different?’ #71
Reassurance	Seeks validation for action or situation	15 (11.5%)	‘I struggle with finding the right amount to try—am I forcing it on her and causing issues, or would she eat nothing if I didn't try?’ #28
Venting	No question asked, venting frustrations	14 (10.8%)	‘I feel a level of anger and frustration I didn't think was possible. She used to be a really really good eater, this started fairly recently. End of rant’. #48
Affirmation	No questions asked, sharing positive experience	6 (4.6%)	‘… for the first time in her life she not only ate all of her dinner … she actually asked for seconds!’ #63
Informational support		21 (16.2%)	
Growth and development	Seeks information about an issue without asking for guidance	14 (10.8%)	‘Is this some sort of regression? Or is it milestone time?’ #60
Nutritional requirements	Seeks information about an issue without asking for guidance	9 (6.9%)	‘How do you portion your toddlers food?’ #34

*Note*: Each post may contain more than one type of support sought.

The majority of parents were seeking practical support for implementing feeding strategies, for overcoming food refusal. Often, these parents disclosed feeding strategies they have tried and tested with little to no success. Several parents specifically asked what to do when their child still refused food despite using a recommended feeding practice such as the division of responsibility (e.g., parent provide and child decide). Some parents described their frustration in relation to the ineffectiveness of advice provided by health professionals other family members or internet websites. However, parents' expectations of the effectiveness of feeding strategies were often based on limited attempts or immediate consumption of food. Some parents appeared to be conflicted by the perceived effectiveness of recommended feeding practices and the satiety needs of their child, with many concerned their child would go hungry. Parents also sought advice for managing the emergence of difficult mealtime behaviours associated with food refusal, such as persistent requests for restricted or preferred foods, crying and tantrums. Several sought practical strategies for managing mealtime structure, including rules and routines, to overcome food refusal. Meal ideas or inspiration for fussy eaters were sought by one fifth of parents (22.3%). The majority of these meal requests were accompanied by the disclosure of a food refusal situation or desire to expand their child's limited dietary variety or nutritional intake. Many parents requested ‘toddler‐approved’ or ‘toddler specific’ recipes, with almost half (44.8%) explicitly referring to their child as ‘picky’.

Almost half (47.7%) of all posts analysed contained appeals for emotional support from parents experiencing the same situation. Parents sought to normalise their child's fussy eating behaviour by directly asking if their situation was ‘normal’, whereas others asked if anyone else had experienced or dealt with the behaviour before. Many parents sought validation for their parenting decisions or reassurance that fussy eating was a transient phase. Uncertainty in managing fussy eating behaviours was evident, with many parents expressing they were ‘at a loss’, ‘don't know what to do anymore’ or ‘at their wits end’. Some parents used the forum to vent their frustrations at their child's ongoing fussiness, whereas a minority of parents shared positive experiences with overcoming food refusal behaviours and expressed high levels of relief and happiness.

Informational support (16.2%) was the least requested type of support sought by parents regarding fussy eating. Parents either sought to identify if their child's fussy eating was a developmental phase or to seek guidance on children's nutritional requirements such as portion sizes, feeding schedules and nutrient requirements.

## 4 DISCUSSION

This study provides a unique insight into parents' concerns about fussy eating in toddlers, through an analysis of support seeking questions posted on the social media platform Reddit. Although previous studies have examined parents' perceptions and management of fussy eating behaviour (Wolstenholme et al., 2020), this is the first study to analyse parents' presentations of fussy eating using an online discussion forum. As such, this study revealed new insights into parents' feeding concerns they may not have felt comfortable articulating elsewhere (Pedersen & Lupton, 2018). Additionally, this study extends previous qualitative studies (Harris et al., 2018; Rubio & Rigal, 2017), by identifying parents' feeding support needs.

As found in previous qualitative studies, our study highlighted the disruptive nature of fussy eating on parents' emotional state and mealtime climate (Harris et al., 2018; Rubio & Rigal, 2017; Trofholz et al., 2017). Frustration and uncertainty were pervasive in parents' concerns and questions regarding fussy eating. Many parents sought support to manage or understand their child's refusal to eat specific foods or meals prepared. Similar to previous studies (Wolstenholme et al., 2020), parents resorted to using coercive (e.g., pressure and punishment) and indulgent (e.g., catering and rewards) feeding practices to manage fussy eating behaviours. Although well intentioned, these practices ultimately limit children's exposure to and acceptance of a variety of foods (Birch et al., 1995).

Concerns for the perceived nutritional inadequacy of children's dietary intakes were common and consistent with prior studies (Harris et al., 2018; Rubio & Rigal, 2017). However, this study found that parents' preoccupation with their child consuming a healthy and balanced diet was often conflicted by their desire for their child to ‘eat something’. This discordance often resulted in the provision of less nutritious foods or catering to children's preferences. Although parents have previously been found to prioritise eating over liking (Goodell et al., 2017; Rubio & Rigal, 2017), this study identified that despite awareness of *what* to feed, parents lacked the knowledge and skills to effectively implement responsive feeding practices. Parents' general awareness of children's nutritional requirements may be influenced by the pervasiveness of public health campaigns emphasising the health benefits of adequate fruit and vegetable consumption (Birch & Fisher, 1998). Findings from this study suggest parents would benefit more from public health messages or guidelines promoting *how* to feed toddlers to establish life‐long healthy eating behaviours (Mitchell et al., 2013).

Parental beliefs about the amount of food children should eat perpetuated the use of non‐responsive feeding practices. These findings are in line with a recent study of feeding concerns which highlighted parents' tendency to prioritise satiation over nutritional quality (Harris et al., 2018). In the present study, the limited quantity of food consumed caused high levels of anxiety about children's lack of weight gain. These concerns often persisted despite receiving reassurance from their child's doctor. Fearing their child would ‘starve’, parents conceded to providing nutritionally poor foods. Parental concern for the adverse impact of quantity consumed on child growth is consistent with prior findings (Goodell et al., 2017; Rubio & Rigal, 2017). However, the current evidence linking fussy eating and underweight is unclear (Brown et al., 2016). A reason for parents' continued unease may be due to a lack of understanding of children's typical growth trajectory across childhood (Gregory et al., 2010). During toddlerhood, children's appetites may appear erratic, due to slower rates of growth and lower energy requirements compared to infancy (Brown et al., 2016; Byrne et al., 2017). Alternatively, it may stem from the strong emphasis health professionals place on monitoring growth and weight gain in infancy. This suggests that acknowledging parental weight concerns and providing evidence‐based information may be important for future feeding interventions.

Another finding from this study is that many parents expressed negative emotions at repeated unsuccessful feeding attempts despite trying multiple strategies, including feeding practices recommended by health professionals (e.g., repeated exposure). Parents perceived that responsive feeding practices were ineffective based on their children's short‐term intake of food or limited attempts. This is despite evidence that children may require up to 10–15 exposures to increase liking for, and consumption of, a previously disliked food (Wardle et al., 2003). Parents' reluctance to repeatedly offer a previously disliked or rejected food has been demonstrated previously, with many parents not persisting beyond five attempts (Carruth et al., 2004). This may indicate that parents have unrealistic expectations of how quickly responsive feeding practices will address fussy eating.

In addition to exploring parents' fussy eating concerns, this study significantly adds to our understanding of the types of support parents are seeking. Almost two thirds of parents sought practical advice (e.g., what and how questions) for managing food refusal behaviours or inadequate intake (quality and quantity). Whereas previous studies have indicated that parents lack awareness of responsive feeding practices (Peters et al., 2012; Rubio & Rigal, 2017; Spence et al., 2016), the present study found parents lacked the practical skills and confidence to implement them effectively. Many parents sought advice on *what* to do when their child still refused food or guidance on *how* long it would take to be successful. As found in previous studies (Brady & Guerin, 2010; Porter & Ispa, 2013), experiential knowledge was highly valued, particularly when health professional advice was deemed ineffective or impractical. This finding supports previous claims that parents lack tangible advice and skills to effectively implement feeding practices (Mitchell et al., 2013; Rubio & Rigal, 2017). Parents also sought practical support in the form of recipes or meal ideas to overcome their child's fussy eating behaviours. Parents' desire for recipes has been observed in a recent evaluation of a child feeding intervention (Haycraft et al., 2020). Although not a new finding, in this context, it may suggest that parents believe fussy eaters like and accept the same foods and are seeking a quick solution to their feeding concern.

In addition to practical advice, parents sought emotional support from other people experiencing the same phenomena. This supports previous findings (Rathbone & Prescott, 2019) that parents seek comfort and reassurance in knowing their child's behaviour is developmentally normal. However, the desire to normalise children's eating behaviour may indicate that parents are unaware that fussy eating is a common developmental phenomenon associated with emerging autonomy (Harris et al., 2018). Additionally, this study found that parents sought reassurance for the decisions and strategies they used to manage fussy eating. These findings highlight parents' uncertainty with navigating feeding and eating experiences during normal and transient phases of child development. Informational support was sought to a lesser degree which suggests parents are mainly using this online forum to seek first‐hand knowledge and experiential advice. The overwhelming requests for practical support may indicate that parents are seeking factual feeding information from other sources such as health professionals, family and friends, websites or books (Mitchell et al., 2013).

### 4.1 Practical implications

Future toddler feeding interventions should focus specifically on fussy eating and parental support for emerging food autonomy during children's formative years (Boswell et al., 2019). These interventions should inform parents and provide reassurance of developmentally appropriate feeding behaviours. Support should also be provided to help parents to understand healthy child growth and appetite during toddler years (Byrne et al., 2017). Supporting parents to establish realistic expectations for measuring the perceived successfulness of responsive feeding practices should be an essential component of feeding interventions or health practitioner consultations. This is of particular importance, as parents are more likely to prioritise satiation over nutritional quality (Harris et al., 2018). Feeding interventions should include practical support for parents to implement feeding practices that promote healthy eating habits. A greater focus on *how* to implement and *what to do* when a child still exhibits fussy eating behaviour would be particularly beneficial.

Nutrition education for health professionals, such as doctors and maternal and child health nurses (MCH), should focus on ‘how to feed’ children using responsive feeding practices. This is especially pertinent as parents frequently access healthcare services in a child's first 5 years of life (Queensland Government, 2020). Additionally, MCH nurses in Melbourne, Australia, have previously been found to focus on ‘what to feed’ in infant feeding consultations rather than advising parents of responsive feeding practices (Laws et al., 2015).

In future, research analysing the types of advice parents receive in response to their support seeking posts may provide valuable insights into the feeding practices parents advocate. Researchers have expressed the challenge online forums present as they position parents as experts and often result in the sharing of misinformation or normalisation of health issues (Appleton et al., 2014, 2017). In this case, parents may be inadvertently normalising coercive or indulgent feeding practices that reinforce undesirable eating behaviours. This is of particular importance as the notion of revered experiential advice in online parenting forums over professional advice has been previously reported (Appleton et al., 2014; Doyle, 2013; Porter & Ispa, 2013).

### 4.2 Strengths and limitations

The method of data collection used in this study has unique strengths. Online forums are widely accessible (24/7) due to ubiquitous internet access and allow parents from socially or geographically distinct areas to interact and share common experiences (Appleton et al., 2014), with immediate responses to their questions (Baker & Yang, 2018). Unlike other social media platforms (e.g., Facebook or Twitter), Reddit forums are popular for their anonymity and unrestricted word counts, thus providing opportunities for more open and honest discourses (Chew et al., 2019) and minimal social desirability bias. Despite the novelty of this study, several limitations need to be acknowledged. This study reflects the feeding concerns of parents during a given 12‐month sampling period. However, the frequency of common fussy eating concerns raised in parents' posts is likely to be an accurate representation of activity within the r/Toddlers subreddit. Additionally, it is the researcher's impression that the parents in this forum were mainly from the United States as evidenced by Reddit usage reports (Statista, 2020) and use of certain phrases (e.g., PP&J sandwiches) or spelling (e.g., paediatrician). A further limitation of analysing posts from online discussion forums such as Reddit is the inability to describe the sociodemographic characteristics of the sample and understand the potential generalisability of findings. It is possible that the parents in the r/Toddlers forum were not representative of people from areas of higher social disadvantage where internet access may be limited. Future research with parents from disadvantaged areas is warranted as they are more likely to experience higher levels of fussy eating (Cardona Cano et al., 2015). Although, it is plausible that parental concerns for fussy eating are universal (e.g., cross‐cultures, social status and education) due to the typical developmental nature of fussy eating in early childhood.

## 5 CONCLUSION

This study contributes to the current knowledge base of fussy eating and provides new insights into parents' feeding concerns and support needs using a novel approach. This study identified that parents appear to be seeking practical solutions to managing common feeding concerns associated with normal developmental stages of toddlerhood. Future feeding interventions should specifically target fussy eating and provide parents with practical management strategies that support them to adopt and maintain responsive feeding practices. Opportunities exist for health professionals to identify feeding concerns and provide practical feeding support at key developmental ages during child health consultations, along with reassurance regarding child appetite regulation and normal growth trajectories. Improving parents' knowledge and skills to manage emerging feeding concerns during toddlerhood may mitigate stressful mealtimes and reduce maladaptive feeding practices that promote obesogenic eating behaviours.

## CONFLICTS OF INTEREST

This project was self‐funded and the authors have no financial relationships relevant to this article to disclose.

## CONTRIBUTIONS

All authors contributed to the research design. BM acquired the dataset. The analysis was conducted by KF with input from RL, NB and BM. KF led the drafting of the manuscript with input from RL, NB and BM. All authors have read and approved the final version of this manuscript.

## Data Availability

The data that support the findings of this study are openly available. A small sample of the Pushshift Reddit dataset are in Zenodo (at http://doi.org/10.5281/zenodo.3608135). The full dataset can be downloaded from https://files.pushshift.io/reddit/submissions for posts and https://files.pushshift.io/reddit/comments for comments.
